# Relationship of SNP rs2645429 in Farnesyl-Diphosphate Farnesyltransferase 1 Gene Promoter with Susceptibility to Lung Cancer

**DOI:** 10.1155/2018/4863757

**Published:** 2018-03-22

**Authors:** Mehdi Dehghani, Zahra Samani, Hassan Abidi, Leila Manzouri, Reza Mahmoudi, Saeed Hosseini Teshnizi, Mohsen Nikseresht

**Affiliations:** ^1^Hematology and Medical Oncology Department, Hematology Research Center, Shiraz University of Medical Sciences, Shiraz, Iran; ^2^Cellular and Molecular Research Center, Yasuj University of Medical Sciences, Yasuj, Iran; ^3^Social Determinant of Health Research Center, Yasuj University of Medical Sciences, Yasuj, Iran; ^4^Biostatistican, Molecular Medicine Research Center, Hormozgan University of Medical Sciences, Bandar Abbas, Iran

## Abstract

**Background and Purpose:**

The mevalonate pathway is one of the major metabolic pathways that use acetyl-CoA to produce sterols and isoprenoids. These compounds can be effective in the growth and development of tumors. One of the enzymes involved in the mevalonate pathway is FDFT1. Different variants of this gene are involved in the risk of suffering various diseases. The present study examined the relationship between FDFT1 rs2645429 polymorphism and the risk of nonsmall cell lung cancer (NSCLC) in a population from southern Iran.

**Method:**

The genotypes of rs2645429 polymorphism of FDFT1 gene were examined in 95 samples: 34 patients with NSCLC and 61 healthy individuals by RFLP method.

**Results:**

The results of this study indicated that C allele of this polymorphism was effectively associated with the risk of NSCLC in the Iranian population (*p* value = 0.023; OR = 2.71; 95% CI = 1.12–6.59) and CC genotype has significant relation with susceptibility to NSCLC (*p* value = 0.029; OR = 3.02; 95% CI = 1.09–8.39). This polymorphism is located in the promoter region FDFT1 gene, and CC genotype may increase the activity of this promoter. This study also found a significant relationship between C allele and metastatic status. C allele was more common in NSCLC patients. (*p* = 0.04).

**Conclusion:**

C allele of FDFT1 rs2645429 polymorphism gene can be a risk factor for NSCLC, whereas T allele probably has a low protective role.

## 1. Introduction

Nonsmall cell lung cancer (NSCLC) is lethal cancer in which approximately half of the patients show metastasis [1, 2]. Lung cancer in the western hemisphere is the second common cancer and is more common among men than women after breast cancer [3, 4]. Lung cancer mortality is higher in developed countries compared to less developed ones and is higher in men than in women. In the world, the incidence of lung cancer in North America is higher than that in the rest of the world, and East Asia ranks fifth worldwide [5]. In Iran, lung cancer is among the five common cancers [6]. The difference in the incidence of lung cancer in different parts of the world can be due to environmental factors and genetic diversity. Genetic variations in different genes can affect the risk of lung cancer [7–14].

One of the hallmarks in different kinds of human cancers is alteration in metabolic pathways such as carbohydrate, lipid, and protein metabolism. The mevalonate pathway is one of these pathways that biosynthesize sterols and isoprenoids [15]. The first reaction of this pathway is the forming of 3-hydroxy-3-methylgrutaryl coenzyme A (HMG-CoA) from the condensation of acetyl-CoA with acetoacetyl-CoA. Then, HMG-CoA was reduced to mevalonate by the HMG-CoA reductase (HMG-CoAR) enzyme. This enzyme is the rate-limiting enzyme for the pathway. This pathway has been studied in various cancers, including breast cancer, multiple myeloma, myeloid leukemia, pancreatic cancer, and liver cancer, and the role of various genes of this pathway has been demonstrated in the molecular mechanism of the mentioned cancers [16–22]. Recent studies showed that the inhibitors of HMG-CoAR that called statins could contribute to the treatment of cancers such as ovarian, breast, and lung cancer [23–25]. The mevalonate pathway can be effective in various cellular processes. Two intermediate products of this route are farnesyl-diphosphate (FPP) and geranylgeranyl diphosphate (GGPP) that participate in the protein prenylation. This is a posttranslational modification of proteins and can affect cell functions such as growth, differentiation, and tumor formation. Many small guanosine triphosphate hydrolases (GTPases) that participate in tumorigenesis, such as RAS and RHO proteins, are isoprenylated, and the inhibition of this pathway can reduce the isoprenylation of these small GTPases. This process can induce the death of some cancer cells [26–31].

FPP is converted into squalene by squalene synthase (SQS). The gene encoding SQS enzyme is farnesyl-diphosphate farnesyltransferase 1 (FDFT1) with the chromosomal position of 8p23. The product of this gene weighs 47 kDa and plays an important role in the production of mevalonate-pathway processes, either from sterol or from the nonsterol route. This gene is expressed in all body tissues, especially in the liver and hypothalamus. This gene has several isoforms; the most common of which has 8 exons, and the promoter of this gene contains sterol regulatory element- (SRE-) like regions [32, 33].

**Figure 1:**
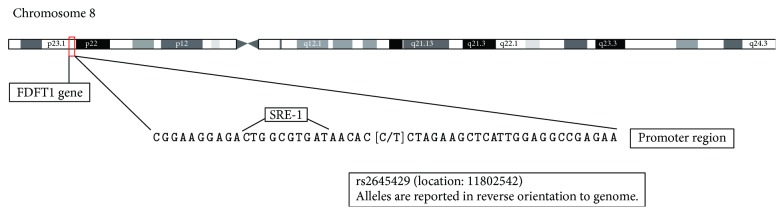
Location of the rs2645429 promoter SNP in the FDFT1 gene.

The genetic diversity of FDFT1 gene has been studied in a number of diseases, including cancers. In investigating the SNP rs2645424 polymorphism of this gene in hepatitis C patients, it was found that this polymorphism was associated with advanced fibrosis in patients with a nonfatty liver [34]. In prostate cancer, the association of the rs2645429 polymorphism of FDFT1 gene has also been observed with progression and invasive phenotypes of this cancer. FDFT1 rs2645429 polymorphism, where nucleotide T replaces C (rs2645429 alleles are reported in reverse orientation to the genome), is located on the promoter of FDFT1 gene (Figure 1). FDFT1 rs2645429 is located at 6 bp upstream of a putative SRE-1, and it has been suggested that the promoter activity of this gene is affected by the replacement of one base in this polymorphism [35]. Given the mentioned points and the importance of the mevalonate pathway in the onset and development of cancer, the present study was designed to determine the relationship between NSCLC and FDFT1 rs2645429 polymorphism gene for the first time.

## 2. Materials and Methods

Thirty-four patients with NSCLC and 61 healthy subjects as the controls were enrolled. The control group was matched to the case group in terms of age and gender. The control group was the people whose pathological tests were negative for lung cancer and who had no history of other cancers, cardiovascular disease, or cholesterol-lowering drugs. The case group was those whose lung cancer, not secondary cancer, was confirmed to be NSCLC by an oncologist, and in fact, results showed no metastases of other nonlung cancers, and they did not have any other chronic disease at the same time. Sampling from the case was done with informed consent and voluntarily. For this purpose, 3 ml of the whole blood was taken from each case to extract DNA. Clinical information of the patients, such as metastasis, stage of disease, and response to treatment was recorded.

DNA was extracted using QIAamp DNA Mini (QIAgen Inc., Santa Clarita, CA). In summary, 100 *μ*l of the whole blood was transferred to a 1.5 ml microtube to extract DNA. Then, 400 *μ*l of lysis buffer was added and vortexed for 20 seconds. Then, 300 *μ*l of precipitant solution was added to the microtubes containing blood and lysis buffer, and vertex was performed for 5 seconds. All of the above solution (800 *μ*l) was transferred to the separator column, and then, the column was centrifuged for 12 minutes at 12000*g* for one minute, and after centrifugation and washing with solutions 1 and 2 and centrifugation in 12000*g*, DNA was extracted by means of an elution buffer and in accordance with kit instructions.

The PCR-RFLP method was used to study genotypes. Primers needed for PCR were designed by the NCBI primer design and Primer3. The fragment containing SNP rs2645429 polymorphism was amplified using forward 5′-GCTGGACCTGTGGAGTAGGT-3′ and reverse 5′-CTCCTGCGCATCCTAAGC-3′ primers. The restriction endonuclease enzyme suitable for the rs2645429 polymorphism was identified by Geneious Basic 4.8.5 software; XbaI (Thermo Fisher Scientific Co., USA) was purchased for this purpose.

After DNA extraction using the primers designed, the PCR test was conducted for all the samples. The steps of performing PCR in brief are 3 minutes at 95°C and 45 cycles for 30 seconds at 95, 58, and 72°C, respectively, and 3 minutes at 72°C. The product of this PCR was 376 bp long and was visible by electrophoresis of 1.5% agarose gel. The samples were then incubated for 16 hours at 37°C by the XbaI restriction enzyme until digested. After incubation, the products were electrophoresed on 2% agarose gel.

Statistical analyses were performed using SPSS 23. *t*-test and chi-square tests were used to compare the means and to examine the relationship between the desired polymorphism genotypes and lung cancer, respectively. In all results, the significance level was considered *p* < 0.05.

## 3. Results

### 3.1. Study Characteristics

The demographic characteristics of the controls and NSCLC cases are presented in Table 1.

The results of this study showed that the variable age followed the normal distribution (*p* = 0.052). There were no significant differences in the frequency distribution of gender in the patient and healthy groups. Although NSCLC was more common in women, this difference was not significant (*p* = 0.06).

### 3.2. Genotypes and NSCLC Risk

**Figure 2:**
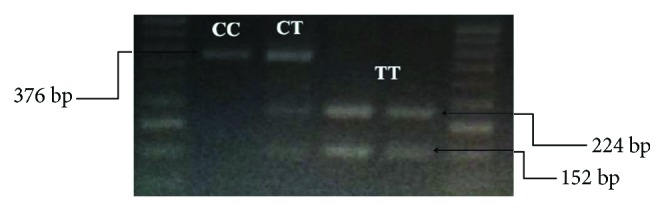
PCR product agarose gel electrophoresis after digestion with XbaI restriction enzyme.

In determining the genotype of the FDFT1 gene, the existence of only one 376 bp band shows homozygous CC genotypes, three bands with lengths of 376, 224, and 152 represent heterozygous CT, and the presence of two bands with lengths of 224 and 152 represents the homozygous TT genotype (Figure 2).

The rs2645429 genotype distributions in the control group were compatible with the Hardy-Weinberg equilibrium (*p* = 0.76).

The average efficiency of the extracted DNA was 5.5 *μ*g/100 *μ*l, and the optical absorption ratio 260/80 was equal to 1.68–1.92.

**Figure 3:**
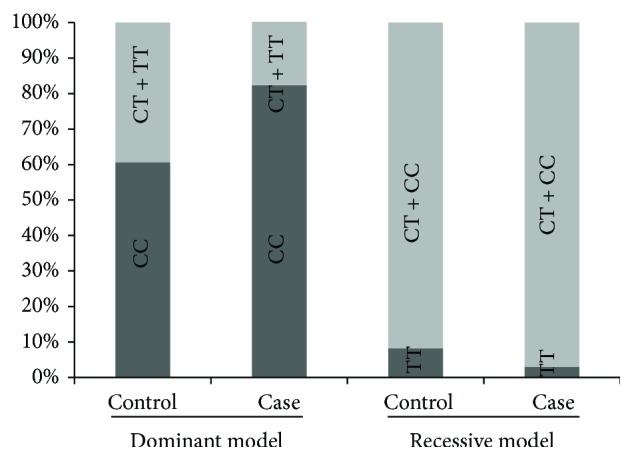
Genotype distribution in case and controls for rs2645429 site. CC genotype frequency was significantly higher in the NSCLC case than the control group under dominant model (*p* value = 0.029; OR = 3.02; 95% CI = 1.09–8.39). CC + CT versus TT was not significant (*p* value = 0.31).

In the present study, 6 (6.3%) subjects had TT genotype, 24 (25.3%) subjects had CT genotype, and 65 (68.4%) subjects had CC genotype (Table 2; Figure 3).

The frequency distribution of the CC genotype was significantly different in the control and case groups (*p* = 0.029). In the case group, 28 cases were CC, five cases were CT, and one case was TT genotypes, and in the control group, five persons were TT, nine persons were CT, and 37 persons were CC genotypes.

There was no significance associated with disease stages and all genotypes (*p* = 0.10). However, those with the CC genotype were at stage IV of cancer. The frequency distribution of C allele was significantly correlated with metastatic status (*p* = 0.04) Table 3.

## 4. Discussion and Conclusion

In the present study, for the first time, we examined the SNP rs2645429 genotype from the FDFT1 gene in patients with NSCLC. The study revealed that C allele could be a risk factor for this cancer.

One of the prominent features of cancer is a change in the metabolic structure of the cells. To support cell proliferation, tumor cells are able to alter the metabolism of carbohydrates, lipids, and proteins. Although most studies have investigated the disorder in regulating carbohydrate metabolism in cancer, recent studies have shown that lipid metabolism can also be associated with the progression of cancer [36–38]. For example, an increase in LDL cholesterol in ovarian cancer patients has been associated with their prognosis [39].

Lung cancer is a global health problem with more than 1.6 million cases a year. It is the second most common cancer and one of the main causes of death from cancer in men and women. Most patients are diagnosed at the advanced level of the disease (III, IV), where the average survival is about 8 to 12 months. There are three types of this cancer, 85% of which are nonsmall cell, 10 to 15% of small cell type, and less than 5% of lung carcinoid tumor type [4, 40].

There are many studies in relation to cholesterol and the risk of lung cancer and its implications; for example, Kucharska-Newton et al. found that the incidence of this cancer was higher in smokers compared to nonsmokers with low levels of HDL cholesterol [41]. In a study by Li et al. [42], it was shown that measuring cholesterol levels before treatment was used as a new independent predictor of NSCLC. In the study of Wu et al., the relationship between serum cholesterol and drug resistance in patients with pulmonary adenocarcinoma has been observed [43].

Given the above data, the importance of the mevalonate pathway in different types of cancers, including lung cancer, was shown. Moreover, it is important to study the role of enzymes and other factors involved in this pathway at different genetic and protein levels. In this study, one of the SNP polymorphisms of FDFT1 (rs2645429) was studied. Stättermayer et al. worked on two rs2645424 polymorphisms of FDFT1 gene and rs738409 of adiponutrin gene (PNPLA3) in patients with chronic hepatitis C, and the association of FDFT1 polymorphism with advanced fibrosis was observed in this disease, which probably resulted from the metabolic changes that this polymorphism can produce in the mevalonate pathway [34]. In the present study, a significant correlation was found between the presence of the CC genotype of FDFT1 gene and the risk of NSCLC with a chance ratio of 3.02 (*p* = 0.029). This finding may indicate C allele being a risk factor for NSCLC.

Schneider et al. studied GSTM1, GSTP1, or GSTT1 gene polymorphism in Caucasian smokers with lung cancer, where there was no significant association between the risk of this cancer and these polymorphisms. Thus, different genotypes of these genes do not play a decisive role in smokers suffering lung cancer [44]; while in the present study, the polymorphisms in FDFT1 gene could be associated with the risk of NSCLC.

Yang et al. [45] conducted a meta-analysis study on 23 studies with a population of 9815 people with lung cancer. There were no significant relationships between the risk of lung cancer and myeloperoxidase- (MPO-) 463G>A [45]. This is contrary to the present study that suggests that the presence of C alleles from FDFT1 gene polymorphism in individuals may increase the risk of lung cancer.

In this study, 19 patients, whose lung cancer stages had been determined based on pathologic findings and the approval of an oncologist, were examined for association with FDFT1 gene rs2645429 polymorphism genotypes. It was observed that individuals who had the C allele of this gene were at more advanced stages of cancer but there was no significance (*p* = 0.26), and this may indicate that C allele increases not only the risk of lung cancer but also the chance of progression. However, due to the small number of samples, this can be checked out more. In other studies, the importance of the polymorphism of some genes in the progression of cancer has been studied; for example, Javid et al. [46] observed that CASP3 (-1337C>G) polymorphism is likely to play a role in the development of NSCLC.

There are several studies which have also shown that SNP polymorphisms can be effective in the advancing and development of other cancers [47, 48] and the present study. These studies indicate the importance of examining the association of the polymorphism of some genes with the progression of cancer.

The results of this study showed that the rs2645429 polymorphism of FDFT1 gene could be associated with the risk of NSCLC, and C allele was considered a risk factor for lung cancer in the patient group. C allele of this gene is also associated with more advanced stages of this cancer, which may indicate that C allele increases not only the risk of lung cancer but also the chance of progression.

Although smoking is a major contributor to lung cancer, due to an increase in the incidence of lung cancer in nonsmokers, it is very important to study other risk factors. As previously mentioned, the alterations in lipid metabolism pathways were shown in different kinds of cancer, such as lung cancer. The cholesterol biosynthesis pathway in relation to cancer has been very much considered. The present study is the first study about one of the other risk factors in the cholesterol biosynthesis pathway in relation with NSCLC, and it is a preliminary study, and to understand how this polymorphism affects lung cancer, we should study more at the function of all genotypes of FDFT1 rs2645429 polymorphism.

## Figures and Tables

**Table 1:** Demographic data for healthy controls and NSCLC patients.

Demographic data	Healthy controls Number (%)	NSCLC patients Number (%)	*p* value
Samples	61	34	—
*Age (years)*			
Male	65.44 ± 7.91	64.25 ± 12.05	0.052^a^
Female	61.59 ± 13.55	55.07 ± 12.43	
*Gender*			0.06^b^
Male	39 (64)	15 (44)	
Female	22 (36)	19 (56)	
*Smoking status*			0.79^b^
Smoker	25 (65.9)	13 (34.1)	
Nonsmoker	36 (63.2)	21 (36.8)	

^a^Based on independent sample *t*-test. ^b^Based on chi-square test. Statistically, there was no difference between age and sex of the healthy and NSCLC groups (*p* > 0.05), but the number of smokers of the healthy control group was significantly higher than that in the NSCLC patient group (*p* = 0.005).

**Table 2:** Genotypic and allelic frequency of FDFT1 polymorphisms in NSCLC patients and control group.

	Control (*N* = 61)	NSCLC (*N* = 34)	OR (95% CI)	*p* value
Age (years)	62.32 ± 4.12	61.08 ± 7.93		
*Gender*				0.46^a^
Male	39 (64)	15 (44)	0.71 (0.28–1.77)	
Female	22 (36)	12 (56)	Reference	
*Smoking status*				
Smoker	25 (65.9)	13 (34.1)	0.89 (0.38–2.11)	0.79^a^
Nonsmoker	36 (63.2)	21 (36.8)	Reference	
*Codominant*				
CC, *n* (%)	37 (60.65)	28 (82.35)		0.09^a^
CT, *n* (%)	19 (31.15)	5 (14.7)		
TT, *n* (%)	5 (8.2)	1 (2.95)		
*Dominant*				
CC, *n* (%)	37 (60.65)	28 (82.35)	3.02 (1.09–8.39)	0.029^a^
CT + TT, *n* (%)	24 (39.35)	6 (17.65)		
*Recessive*				
TT, *n* (%)	5 (8.2)	1 (2.95)	0.33 (0.03–3.03)	0.31^a^
CT + CC, *n* (%)	56 (91.8)	33 (97. 05)		
*Allele*				
C, *n* (%)	93 (76.23)	61 (89.7)	2.71 (1.12–6.59)	0.023^a^
T, *n* (%)	29 (23.77)	7 (10.3)	Reference	

^a^Based on chi-square test.

**Table 3:** Association between FDFT1 gene polymorphism and clinicopathological characteristics of Non-small Cell lung cancer.

Clinical characteristics	Genotype (*N* = 19)			Allele (*N* = 38)	
*Clinical staging*	CC	CT	TT	T	C
Stage IIIA, IIIB	3 (15.79)	0 (0.0)	1 (5.26)	2 (5.26)	6 (15.79)
Stage IV	12 (63.16)	3 (15.79)	0 (0.0)	3 (7.89)	27 (71.05)
Fisher's exact test		*p* = 0.1		*p* = 0.26	
*Metastasis*					
Positive	8 (42.11)	0 (0.0)	0 (0.0)	0 (0.0)	16 (42.1)
Negative	7 (36.84)	3 (15.79)	1 (5.26)	5 (13.16)	17 (44.74)
Fisher's exact test	*p* = 0.15			*p* = 0.04^∗^	
*Treatment*					
Response	8 (42.11)	2 (10.53)	1 (5.26)	4 (10.53)	18 (47.37)
Nonresponse	7 (36.84)	1 (5.26)	0 (0.0)	1 (2.63)	15 (39.47)
Fisher's exact test	*p* = 0.62			*p* = 0.28	
*NSCLC types*					
Adenocarcinoma	11 (57.89)	3 (15.79)	1 (5.26)	5 (13.16)	25 (65.79)
Squamous cell carcinoma	4 (21.05)	0 (0.0)	0 (0.0)	0 (0.0)	8 (21.05)
Fisher's exact test	*p* = 0.5			*p* = 0.21	

^∗^There is a significant correlation between metastatic status and C allele distribution (*p* = 0.04).
